# Prioritizing Tuberculosis Clusters by Genotype for Public Health Action, Washington, USA

**DOI:** 10.3201/eid1903.121453

**Published:** 2013-03

**Authors:** Scott Lindquist, Sheanne Allen, Kim Field, Smita Ghosh, Maryam B. Haddad, Masahiro Narita, Eyal Oren

**Affiliations:** Author affiliations: Kitsap County Health District, Bremerton, Washington, USA (S. Lindquist); Washington State Department of Health, Olympia, Washington, USA (S. Lindquist, S. Allen, K. Field);; Centers for Disease Control and Prevention, Atlanta, Georgia, USA (S. Ghosh, M.B. Haddad);; Public Health–Seattle & King County Tuberculosis Control Program, Seattle, Washington, USA (M. Narita, E. Oren).

**Keywords:** tuberculosis, genotype, disease outbreaks, clusters, tuberculosis and other mycobacteria, United States, Washington, TB, genotyping, Mycobacterium tuberculosis

## Abstract

Groups of tuberculosis cases with indistinguishable *Mycobacterium tuberculosis* genotypes (clusters) might represent recent transmission. We compared geospatial concentration of genotype clusters with independent priority rankings determined by local public health officials; findings were highly correlated. Routine use of geospatial statistics could help health departments identify recent disease transmission.

*Mycobacterium tuberculosis* genotyping has been applied to tuberculosis (TB) control activities for >2 decades, and epidemiologic or genotyping data can confirm or disprove outbreaks (*1*–*4*). Investigation of genotype clusters can identify unrecognized transmission and lead to interventions that interrupt further transmission (*5*,*6*). However, cluster investigations are complex, requiring patient interviews and field observations. Focusing resources on clusters that most likely represent recent TB transmission could reduce the number of unnecessary investigations.

Geospatial statistics can identify higher-than-expected concentrations of TB cases with indistinguishable genotypes (*7*). We describe a comparison of a quantitative geospatial statistic analysis with qualitative expert opinion for prioritizing TB cluster investigations in Washington, USA, a state with moderate TB incidence (3.5 cases/100,000 persons) (*8*). The comparison was performed for initial and follow-up 3-year periods, 2005–2007 (period 1) and 2008–2010 (period 2).

## The Study

TB genotype clusters were defined as groups of >3 TB case-patients whose isolates had matching spoligotyping and 12-locus mycobacterial interspersed repetitive unit–variable number tandem repeat (MIRU-VNTR) (*9*) genotyping results that were reported in the same county within Washington. A log-likelihood ratio (LLR) was calculated for each genotype cluster identified during each of the two 3-year periods (Figure). The larger the LLR, the greater the possibility the cluster represented geographically concentrated TB cases, a proxy for recent TB transmission. The cutoff point for the LLR was set to 5.0, based on the value used by the national TB Genotyping Information Management System (*10*).

**Figure:**
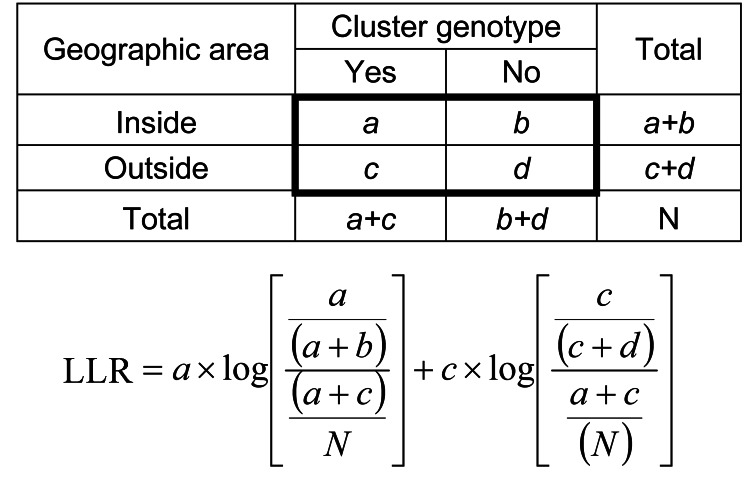
Formula used to calculate geospatial statistic (a modified log-likelihood ratio [LLR]) on the basis of geographic distribution of *Mycobacterium tuberculosis* genotype clusters, Washington, USA. Variables are classified as follows: *a* = number of tuberculosis (TB) cases with the genotype of interest in the selected county; *b* = number of cases with the genotype of interest in the United States; *c* = number of cases without the genotype of interest in the selected county; *d* = number of cases without the genotype of interest in the United States; *N* = total number of TB cases.

Qualitative analysis came from a 5-member expert panel of TB public health officials in Washington. In 2008, the panel participated in a discussion of all county-level TB clusters, ranking each as high or low priority for additional investigation. Priority was determined on the basis of a review of patient characteristics, epidemiologic links from field investigations, and maps of genotype distributions. The panel also had information from enhanced contact investigations from local public health investigation teams that included the ability to order IS*6110* restriction fragment-length polymorphism (IS*6110* RFLP) and 24-locus MIRU-VNTR testing for clusters of concern, but results from these tests were not universally available. The ranking exercise with the same 5-member panel was repeated after period 1 for clusters from period 1. The expert panel was blinded to the LLR.

LLRs were compared with the expert opinion ranking to assess concordance. With expert opinion as the standard, negative and positive predictive values (NPV and PPV, respectively) were calculated for period 1 using a cutoff point of LLR >5.0. Alternative cutoff points were evaluated to maximize NPV and PPV. Sensitivity and specificity of the >5.0 LLR cutoff point and exact binomial 95% CIs were calculated for period 1 clusters. An alternative cutoff point to maximize sensitivity and specificity was also determined.

A total of 806 TB cases were reported in Washington during period 1. Of 659 culture-positive cases, 642 (97.4%) had genotyped isolates; of these, 318 cases formed 21 clusters. Five of these clusters had a high LLR; the expert panel ranked all 5 of these clusters high priority and identified them as clusters of concern. Of the 16 clusters with LLR <5.0, the expert panel ranked 12 (75.0%) as low priority (Table).

**Table:** Comparison of geospatial analysis results and expert panel priority status rankings for county-level genotype clusters of TB cases, Washington, USA, 2005–2010*

County	Spoliogtype	12-locus MIRU-VNTR	Period 1†				Period 2†			Key epidemiologic features‡
			No. cases	LLR	Expert priority		No. cases	LLR	Expert priority	
A	000000000003771	223321153643	32	**31.8**	High		19	**18.3**	High	H, SA
	000000000003771	223325173533	17	0.1	High		8	0.4	Low	FB
	677777477413771	254326223432	14	0.2	Low		14	0.1	Low	FB
	703377400001771	227425113434	4	1.2	Low		6	2.9	Low	FB, H
	000000000003771	223325153533	3	0.5	Low		1	<0.01	Low	FB
	000000000003771	223325163533	3	0.2	Low		7	1.7	Low	FB, SA
	677777477413771	254326223422	6	1.3	Low		4	0.4	Low	FB
	777000377760771	225125113322	3	0.6	Low		3	1.0	Low	FB
	000000000003771	222325173543	9	2.4	High		3	0.1	High	H, SA
	777777777760771	225125113322	3	0.7	Low		2	0.4	Low	FB
	000000000003771	222325173533	7	2.6	High		8	2.3	High	
	000000000003771	223325173433	3	1.5	Low		1	0.2	Low	FB
	000000000003771	223325133533	3	1.4	Low		0	<0.01	Low	FB
	000000000000000	223325123534	4	**9.2**	High		0	<0.01	Low	H, SA
	777776777620601	224325153323	3	4.4	High		0	0.03	Low	H, SA
B	000000000003771	223425173563	13	**19.5**	High		8	**13**	High	H, SA
C	677777477413771	254326223432	6	1.3	Low		3	<0.01	Low	FB, SA
	777776777760771	125325153225	7	**9.8**	High		2	1.8	High	H, SA
D	677777477413771	254326223432	5	0.8	Low		4	0.4	Low	FB
	777776757760771	223325143324	6	**8.7**	High		1	1.2	Low	
E	677777477413771	254326223432	3	1.0	Low		2	0.9	Low	FB

*LLRs >5.0 (**boldface**) indicate greater possibility that the cluster represents geographically concentrated TB cases, a proxy for recent TB transmission). TB, tuberculosis; MIRU-VNTR, mycobacterial interspersed repetitive unit–variable number tandem repeat; LLR, log-likelihood ratio, a measure of geospatial concentration; H, homelessness; FB, foreign born; SA, substance abuse.  †Period 1, January 1, 2005–December 31, 2007; period 2, January 1, 2008–December 31, 2010. ‡Blank cells indicate none present.

A total of 723 TB cases were reported in Washington during period 2. Of 592 culture-positive cases, 576 (97.3%) had genotyped isolates. The expert panel reexamined the 21 clusters identified during period 1 and focused on new activity within those areas during period 2. Two clusters with a high LLR during period 1 continued to have a high LLR during period 2; the expert panel continued to rank these high priority. The 3 other clusters that had a high LLR during period 1 had a low LLR for period 2; one of those was still considered a high priority by the panel. Of the remaining 16 clusters, which continued to have a LLR <5, 14 (87.5%) were still ranked low priority by the panel.

Two clusters in the same county, PCR00309 and PCR00803, had low LLRs but were considered high priority by the expert panel. For cluster PCR00309, the panel cited high levels of homelessness among case-patients as reason to rank it high priority. For cluster PCR00803, the panel cited a highly mobile population from a TB-endemic country that regularly traveled into and out of the United States as reason to rank it high priority. However, the travel history among case-patients in this cluster made it difficult for investigators to determine whether transmission was occurring within Washington or abroad.

The NPV and PPV for a LLR cutoff point of 5.0 were 75% and 100%, respectively. Lowering the cutoff point to a LLR >2.0 increased the NPV to 92.3%, but the PPV remained at 100%. For period 1 clusters only, a cutoff point of LLR >5.0 generated a sensitivity of 55.6% (95% CI 21.2%–86.3%) and specificity of 100% (95% CI 73.5%–100.0%) for identifying clusters for further investigation. Decreasing the cutoff point to >2.0 increased the sensitivity to 88.9% (95% CI 51.8%–99.7%) but did not change the specificity (100%; 95% CI 73.5%–100.0%).

## Conclusions

The geospatial statistic in this study was highly correlated with experts’ perceived need for public health action. This finding indicates that automated alerts generated on the basis of geospatial concentration of TB cases might help the state TB program identify clusters that would benefit from additional investigation. Automated alerts can be generated by using routinely collected surveillance data and are currently part of the national TB Genotyping Information Management System (*10*). 

Patient and contact characteristics, transmission venues, and temporality all contribute toward prioritization determination. For example, during period 1, a total of 6 (66.6%) clusters ranked high priority by the expert panel were characterized by homelessness or substance abuse among case-patients, and 8 (88.9%) were characterized by US-born case-patients (Table). 

Conversely, 11 (91.7%) clusters ranked low priority were characterized by case-patients who were foreign-born, a known risk factor for latent TB infection (*7*). None of the period 1 clusters with LLR >5 and only 1 of 9 clusters ranked as high priority by the expert panel were characterized by foreign-born case-patients. These results indicate the need for further study to identify the limitations of the LLR score in detecting localized and recent TB transmission among foreign-born case-patients. 

The availability of IS*6110* RFLP or 24-locus MIRU-VNTR testing results to the expert panel is the current standard for fieldwork and could have introduced an information bias for the panel in this study. Although this effect is unknown, lack of universal IS*6110* RFLP and 24-locus MIRU-VNTR test results is a limitation of this study.

We found that geospatial statistics based on TB genotyping and surveillance data could help identify and prioritize likely recent disease transmission events in Washington. In addition, LLR values should be incorporated into ongoing evaluation by the expert panel; in fact, LLR is now included in routine genotype and cluster reviews. Geospatial statistics are an attractive approach to prioritization, but additional field-based research is needed to assess whether factors such as epidemiologic characteristics could be used to further develop a prioritization algorithm. Integrating these factors and determining ideal cutoff points in different settings will increase predictive value. 
